# Neuroprotective potential of erythropoietin in neonates; design of a randomized trial

**DOI:** 10.1186/s40748-015-0028-z

**Published:** 2015-12-02

**Authors:** Sandra E. Juul, Dennis E. Mayock, Bryan A. Comstock, Patrick J. Heagerty

**Affiliations:** Department of Pediatrics, Division of Neonatology, University of Washington, 1959 Pacific Ave NE, Box 356320, Seattle, WA 98195-6320 USA; Department of Biostatistics, University of Washington, 4333 Brooklyn Avenue NE, Box 359461, Seattle, WA 98195-9461 USA

**Keywords:** Prematurity, Neuroprotection, ELGANS, Biomarkers

## Abstract

**Background:**

In 2013, nearly four million babies were born in the U.S., among whom 447,875 were born preterm. Approximately 30,000 of these infants were born before 28 weeks of gestation. These infants, termed **E**xtremely **L**ow **G**estational **A**ge **N**eonates (ELGANs), experience high morbidity and mortality despite modern therapies: approximately 20 % of ELGANs admitted to an NICU die before discharge, 20 % of survivors have severe, and 20 % moderate neurodevelopmental impairment (NDI). New approaches are needed to improve neonatal outcomes. Recombinant erythropoietin (Epo) is a promising neuroprotective agent that is widely available, affordable, and has been used safely in neonates to stimulate erythropoiesis. There are extensive preclinical data to support its use as a neuroprotective intervention: Epo promotes normal brain maturation by increasing neurogenesis, angiogenesis, and by protecting oligodendrocytes. Epo also decreases acute brain injury following hypoxia ischemia by decreasing inflammation, oxidative and excitotoxic injury, resulting in decreased apoptosis. Despite the availability of both preclinical and safety data there has not been a definitive clinical evaluation of the benefit of Epo, and a large phase III trial is necessary to provide evidence to support potential changes in practice guidelines.

**Findings:**

We first review the preclinical data motivating further clinical trials, and then describe in detail the design of the PENUT study (**P**reterm **E**po **N**e**u**ropro**t**ection). PENUT is a phase III study evaluating the effect of neonatal Epo treatment on the combined outcome of death or severe NDI among ELGANS. 940 subjects will be randomized to determine: 1) whether Epo decreases the combined outcome of death or NDI at 22–26 months corrected age; 2) the safety of high dose Epo administration to ELGANs; 3) whether Epo treatment decreases serial measures of circulating inflammatory mediators, and improves biomarkers of brain injury; and 4) whether Epo treatment improves brain structure at 36 weeks postmenstrual age as measured by MRI.

**Conclusions:**

Epo neuroprotection is an exciting new approach to preterm neuroprotection, and if efficacious, will provide a much-needed therapy for this group of vulnerable infants.

## Introduction

**E**xtremely **L**ow **G**estational **A**ge **N**eonates (ELGANs) are at high risk of death or neurodevelopmental impairment (NDI). Data from 9575 ELGANs born between 2003 and 2007 and admitted to Neonatal Research Network intensive care units showed that death or NDI occurred in 91, 80, 66 and 56 % of those born at 24, 25, 26 and 27 weeks gestation, respectively [1]. These sobering statistics do not include infants that died before admission to a NICU, or those who died within 12 h of admission. Major neurologic morbidities, which include cerebral palsy (CP), deafness, blindness, and cognitive disabilities, are present in up to 50 % of surviving extremely preterm infants at school age [2–7]. In addition to the traditional measures of impairment, long-term follow-up studies are now also increasingly identifying behavioral dysfunctions such as attention deficit disorder and autism spectrum disorder [8–11]. Sequelae of extreme prematurity are a tremendous burden to the individuals, their families, and to our health care system, accounting for nearly half of the health care dollars spent on newborn care [12]. Clearly, a neuroprotective intervention that improves outcomes for ELGANs would be profoundly beneficial to the individual, the family and to society [13].

## Findings

A phase III study to test the safety and efficacy of high dose Epo is indicated, based on current preliminarypreclinical and clinical data.

The PENUT (Preterm Epo Neuroprotection) Trial is a randomized, placebo controlled, double blind study ofEpo neuroprotection in an ELGAN population.

## Review

### Vulnerabilities of the preterm brain

ELGANs are born at a time when the fetal brain is rapidly increasing in size, shape and complexity [14, 15]. Brain development is vulnerable to interruption by hypoxia-ischemia, oxidant stress, inflammation, and excitotoxicity, as evidenced by structural, biochemical, and cell-specific injury [16, 17]. Oligodendrocytes, which emerge and mature between 24 and 32 weeks of development, are particularly susceptible to injury, resulting in the white matter injury (WMI) characteristic of preterm infants [18]. Although the transition from fetal to early postnatal life is the period of greatest vulnerability [19], ELGANs remain at risk for brain injury throughout the period of oligodendrocyte development.

Perinatal inflammation (chorioamnionitis, necrotizing enterocolitis, or sepsis) is associated with increased risk of NDI [2, 20, 21]. Microglial activation [22] and increased cytokine expression, particularly TNF-α, interleukin (IL)-6, and IL-8, have been associated with brain injury in preterm infants [23, 24] and in animal models of neonatal brain injury [25].

### Epo neuroprotection

Epo has anti-inflammatory, anti-excitotoxic, anti-oxidant, and anti-apoptotic effects on neurons and oligodendrocytes, and promotes neurogenesis and angiogenesis, which are essential for repair of injury and normal neurodevelopment [26–30]. Epo effects are dose-dependent, and multiple doses are more effective than single doses [31–33]. Epo reduces neuronal loss and learning impairment following brain injury [34]. Even when initiated as late as 48–72 h after injury, there is evidence of improved behavioral outcomes, enhanced neurogenesis, increased axonal sprouting, and reduced white matter injury [35, 36]. Epo has demonstrated anti-inflammatory effects, which may contribute to neuroprotection in the scenario of preterm birth and increased inflammatory activity [37–43]. Epo also stimulates growth factors required for normal brain growth such as brain-derived neurotrophic factor (BDNF) and glial cell derived neurotrophic factor (GDNF) [27, 44].

Epo decreases WMI in adult and neonatal animal models of brain injury [35, 45–50]. Preliminary work in preterm infants suggests this holds true for developing human brain [51]. This protective effect may be mediated by the effect of Epo on oligodendrocytes: Epo promotes the proliferation, maturation and differentiation these cells [52], and protects them from injury induced by interferon-γ, LPS, and hypoxic-ischemia [35, 53, 54].

In addition to cell specific effects in brain, Epo increases iron utilization as erythropoiesis is increased. Iron is highly reactive and normally sequestered by transport proteins. Unbound iron produces free radicals and subsequent oxidative injury. Preterm infants have measurable free iron, which increases after transfusions of red blood cells or during metabolic instability such as sepsis [55, 56]. Epo may contribute to neuroprotection by decreasing free iron.

#### Epo dosing

**Fig. 1:**
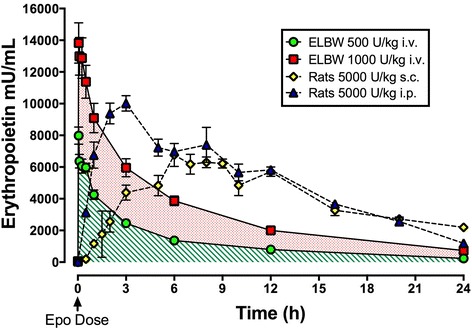
Epo pharmacokinetics in neonatal rats compared to extremely low birth weight infants (ELBW). Epo concentration in mU/mL is shown on the Y axis, and time in hours on the X axis. Serum concentration in neonatal rats is shown following subcutaneous injection (s.c.) or intraperitoneal injection (i.p.) of 5000 U/kg/dose of Epo. This is compared to dosing in human ELBW infants with 500 U/kg/dose or 1000 U/kg/dose by intravenous injection (i.v.). The area under the curve (AUC) of 1000 U/kg/dose most closely approximates the neuroprotective concentrations noted in rats treated with 5000 U/kg/dose

In rodent, ovine and nonhuman primate models of neonatal brain injury, repeated Epo doses of 1000–5000 U/kg/dose result in sustained neuroprotection, improving both short and long-term structure and function [31, 32, 49, 57–59]. Higher doses are needed for neuroprotection than for erythropoiesis, due to the low percentage of circulating Epo that crosses the blood brain barrier [60]. In acute models of brain injury, including a late dose (7 days post injury) significantly improves outcomes [32, 61]. Preclinical data suggest that Epo neuroprotection has a U-shaped dosing curve, with too little or too much Epo resulting in diminished efficacy [31, 62]. To estimate how neuroprotective Epo doses in rat pups relate to human pharmacokinetics, plasma Epo concentrations were measured in extremely low birth weight infants (<1000 g birth weight) after 500, 1000, and 2500 U/kg/dose [63]. Nonlinear kinetics were noted, consistent with previous studies in neonates [64]. In these infants, intravenous administration of 500 and 1000 U/kg resulted in similar peak concentrations but faster clearance than were achieved in rat pups after 5000 U/kg (Fig. 1). Doses of 1000 U/kg Epo resulted in area under the curve (AUC) measurements most similar to the most protective dose in rats [31]. The 500 U/kg dose fell short (one third to one quarter the protective AUC), while 2500 U/kg was close to three times the optimal dose in rats. Minimum steady-state concentrations (mean = 576 mU/ml) were produced using the 1000 U/kg/dose. Thus, we estimate that multiple doses of 1000 U/kg would be safe and achieve neuroprotective circulating concentrations in human neonates.

## Translational trials of neonatal Epo neuroprotection for preterm infants are in progress

Enrollment in a randomized, double masked phase II trial of Epo neuroprotection for preterm infants has been completed (NCT00413946). 443 infants (gestational age 26 0/7-31 6/7 weeks) were randomized to Epo (3000 U/kg, *N* = 229) or saline (*N* = 214) at 3, 12–18, and 36–42 h after birth. This dose and dosing regimen was found to be safe [65], and those treated with Epo showed improved white matter integrity [51, 66]. Long term follow up is ongoing.

Two additional preliminary reports of preterm infants treated prospectively show benefit: 1) Preterm infants 500 to 1250 g treated with either Epo (400 U/kg 3x/week, *N* = 29) or Darbepoetin (10 U/kg/dose once a week, *N* = 27) from birth to 35 weeks postmenstrual age (PMA) had an average cumulative cognitive score 8 to 10 points higher than placebo/controls with Epo-treated infants earning scores of 97.9 ± 14, and Darbepoetin-treated infants scoring 96.2 ± 7.3 compared to 88 ± 14 for controls (*N* = 24). Epo recipients also performed statistically better than controls on object permanence testing [67]. The combined scores for NDI or death were significantly better in both Epo and Darbepoetin treated groups, with combined scores of 15.5 % compared to 48.2 % in the control group. 2) Follow-up of ELBW infants that received 500 to 2500 U/kg Epo x 3 doses in a phase I/II trial [63] showed that Epo treatment correlated with improvement of cognitive (*R* = .22, *p* < 0.05) and motor (*R* = .15, *p* < 0.05) scores [68].

### Risks of intervention

In adults, complications of prolonged Epo treatment include polycythemia, seizures, hypertension, stroke, myocardial infarction, congestive heart failure, tumor progression, and shortened time to death. None of these adverse effects have been reported in Epo-treated neonates in over 3000 patients enrolled in randomized controlled trials [69]. Epo trials in neonates for the purposes of testing its erythropoietic effect have shown it to be a safe drug for use in this population. There is robust data from preclinical animal work showing that Epo, when used at optimal doses (1000–5000 U/kg), shows short and long term improvement in brain injury that approximates 50–80 %, and no safety issues have been discovered. Even the risk of retinopathy of prematurity (ROP) has not been substantiated in randomized controlled trials [70, 71]. Safety data for high dose Epo (3000 U/kg x 3 doses) have recently been published [65]. However, as yet unknown rare complications may occur, so safety data must still be collected as studies of Epo neuroprotection are done.

**Fig. 2:**
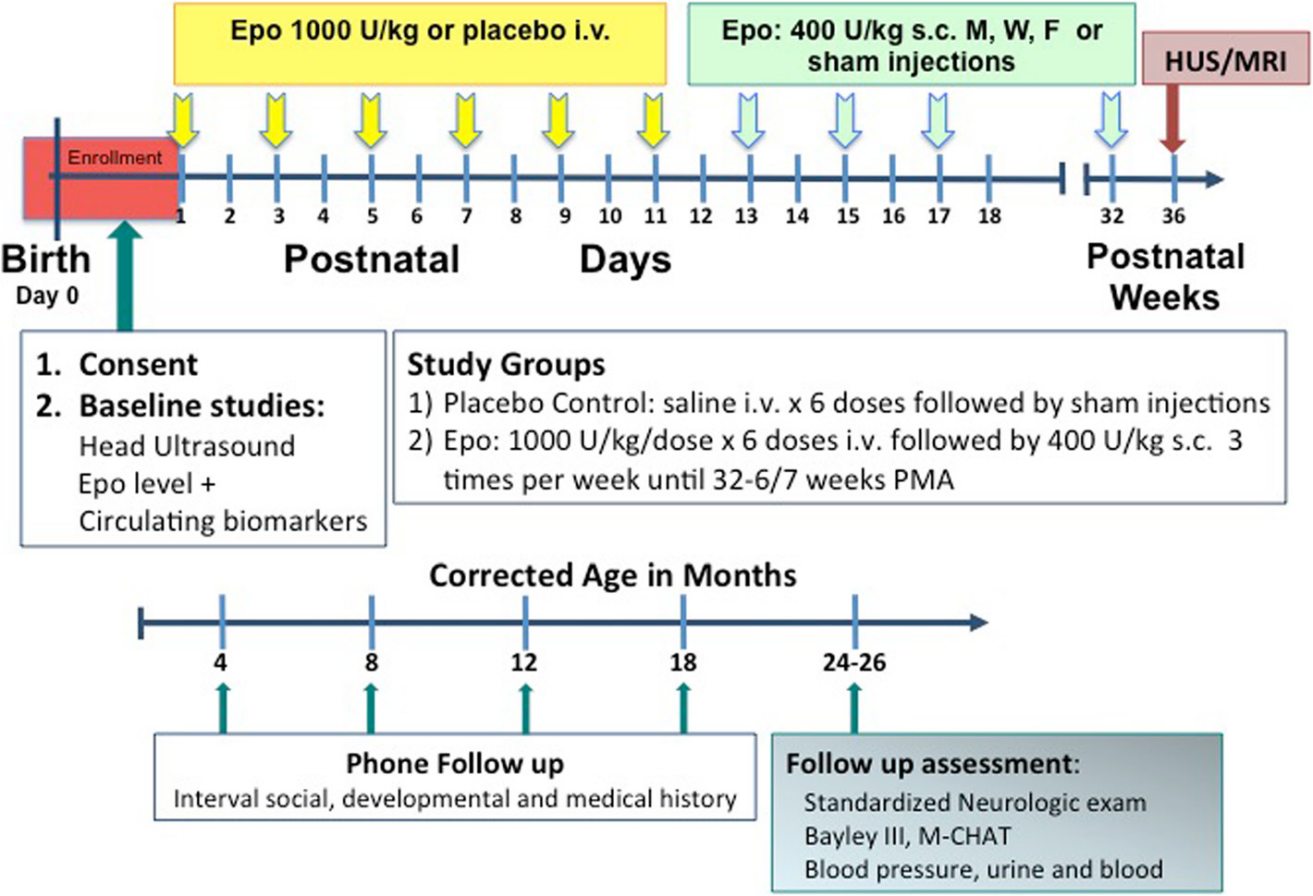
PENUT Trial Overview. Significant events each subject will undergo when participating in the PENUT trial

The **PENUT** (**P**reterm **E**po **N**e**u**ropro**t**ection) **Trial** is a randomized, placebo controlled, double blind study of Epo neuroprotection in an ELGAN population. Figure 2 provides an overview of the study. 940 patients will be enrolled at 19 sites across the United States in order to evaluate 752 infants at 22–26 months corrected age. Enrollment and initial treatment with study drug will occur by 24 h after birth. Subjects will be randomized to either Epo treatment or placebo, and treatment will continue until 32-6/7 weeks PMA. Short term, intermediate and long-term safety measures will be determined by comparing Epo-treated and control infants. Mechanisms of Epo neuroprotection and potential biomarkers of outcome will be sought by measuring sequential inflammatory cytokines and markers of brain injury. In a subset of subjects, a brain MRI will be done at 36 weeks PMA to determine whether Epo treatment preserves brain growth and decreases injury. After discharge from the hospital, phone contact will be made at 4 to 6 month intervals. Data will be collected on interval medical history and functional status. In- person follow-up will occur at two years corrected age (22–26 months), at which time standardized neurodevelopmental assessments will be made. The primary outcome is death or severe NDI at 22–26 months corrected age, with a secondary outcome of death, severe or moderate NDI. This study of high dose Epo for the purposes of neuroprotection of preterm infants is registered with the FDA (IND # 12656) and ClinicalTrials.gov (NCT01378273).

#### Eligibility and enrollment

Patients will be eligible if they are NICU inpatients between 24–0/7 and 27–6/7 weeks of gestation and less than 24 h of age with arterial or venous access. Parental consent may be obtained prenatally or postnatally, as dictated by each recruitment site’s IRB. Patients will be excluded if there are known major life-threatening anomalies, known or suspected chromosomal anomalies, severe hematologic crises such as disseminated intravascular coagulopathy, twin-twin transfusion such that 1 twin is not eligible due to polycythemia or hydrops, polycythemia (hematocrit > 65 %), hydrops fetalis, or known congenital infection such as toxoplasmosis, CMV, rubella or syphilis.

#### Randomization

We will use block randomization within site using variable blocks of size 4 to 10 subjects. Using block randomization ensures that equal numbers of subjects are randomized to the intervention and control arm and that the two groups are balanced at period enrollment intervals. For multiple births (twins, triplets, etc.), all infants will be randomized to the same treatment group (e.g. effective randomization of the mother). Randomization sequences will be provided to the research pharmacy at each site. Randomization will be stratified on site, gestational age category (24–25 weeks, 26–27 weeks), and on multiple gestation (number of babies carried to birth: singleton, twins, triplets, or more).

### Epo dose justification

The Epo dose, and duration of therapy chosen for this study is based upon available preclinical and clinical data for Epo neuroprotection. Although doses as high as 3000 U/kg/dose are being tested in preterm infants without apparent adverse effects [65], we chose 1000 U/kg/dose based on our phase I/II data [63]. We will treat with high dose during the first weeks of life when physiologic vulnerability is highest, followed by maintenance Epo through 32 weeks postmenstrual age (the period of oligodendrocyte vulnerability).

#### Iron supplementation

Maintaining iron sufficiency in a growing preterm infant is important for normal brain growth. Because of this, iron guidelines were established for the PENUT trial. When enteral feedings are started, a standard iron containing formula is used if breast milk is unavailable. Once infants (all subjects) reach an enteral intake of 60 mL/kg/d and are at least one week old, they are started on enteral iron at a dose of 3 mg/kg/d total. Enteral iron is increased to 6 mg/kg/d when infants achieve an enteral intake of 100 to 120 mL/kg/d [72]. Serum ferritin or ZnPP/H ratios are checked at 14 and 42 days, and iron adjusted accordingly. If subjects are not able to tolerate enteral feedings and oral iron supplements, they will be given maintenance iron parenterally (3 mg/kg/week, adjusted based on iron indices).

#### Study procedures

**Fig. 3:**
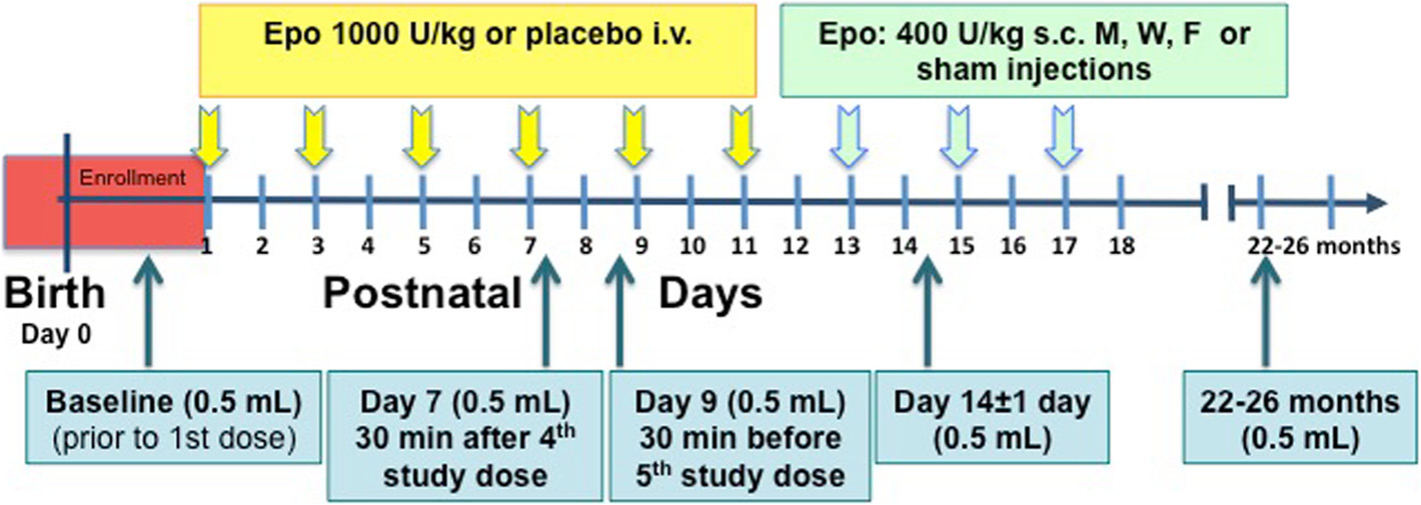
PENUT Trial Blood Draws. The timing of all PENUT related blood draws is shown schematically

Five 0.5 mL blood samples will be drawn from each enrolled subject on the following schedule: prior to the first study drug dose, 30 min after the 4th study drug dose (peak), 30 min prior to the 5th study drug dose (trough), on day 14 ± 1 day, and at the 22–26 month in-person visit (Fig. 3). These samples will be used to determine circulating Epo concentrations, cytokine measurements, and biomarkers of brain injury. The following inflammatory markers and growth factors will be assayed: BDNF, Interferon-gamma (IFN-γ), IL-1β, IL-6, IL-8, IL-10, tumor necrosis factor-α (TNF-α), transforming growth factor (TGF)-β, matrix metalloproteinase (MMP)-2 and MMP-9, macrophage inflammatory protein-1α (MIP-1α), MIP-1β, monocyte chemotactic protein-1 (MCP-1) and tissue inhibitor of metalloproteinase (TIMP)-1 [50, 73–75]. Markers of neurotoxicity and brain injury will include: S100B, glial fibrillary acidic protein (GFAP), neuron specific enolase (NSE), Tau, Activin A, and Ubiquitin C-terminal hydrolase-L1 (UCH-L1) [76–78].

Brain magnetic resonance imaging (MRI) will be obtained on 110 subjects from each study arm at 36 weeks PMA (220 total). All scans will be done on 3 T scanners using an optimized, standardized protocol. MRI’s will be evaluated centrally, and analysis will include both quantitative [79, 80] and qualitative evaluations [81].

#### Outcomes

The primary outcome variable is the composite outcome of death or neurodevelopmental impairment at 22–26 months. All personnel involved in the neurodevelopmental assessments will undergo standardized training, and will be blind to study treatment. Assessment at 22–26 months corrected age will include: Bayley III Scales of Infant and Toddler Development, a standardized neurological examination, Gross Motor Function Classification System (GMFCS) assessment, the Child Behavior Checklist, and the Modified Checklist for Autism in Toddlers (M-CHAT-R). Neurodevelopmental impairment (NDI) is defined as the presence of any one of the following: CP, Bayley III cognitive or motor scale < 70. There is a known inflation of scores from the Bayley II to III [82–84] and we will therefore also consider a threshold of <85 for secondary analysis. Cerebral palsy will be categorized based on features present on standardized neurologic exam, and classified as mild, moderate, or severe, with hemiplegia or diplegia. Two stepped outcomes will be used: the primary outcome is very stringent, and uses a cut off of two standard deviations below the mean for cognitive or motor scales (<70). The secondary outcome uses a cut off of one standard deviation below the mean for these criteria (<85), which will still have a significant impact on the child, family, and healthcare system. The 2-year assessment will provide a window into early language development and early gross- and fine-motor development. We plan to submit further grant applications for long-term follow-up at 5 years of age, which correlates better with ultimate function [85].

#### Power and sample size for primary outcome

In order to determine the necessary sample size for efficacy evaluation, we formulated assumptions for the primary outcome rate in the treated and untreated groups. The primary outcome measure is the rate of death or severe NDI. Using data from two sources, we computed the expected rates of death or NDI for the neonates that we will enroll. The Vermont Oxford Network 2008 Follow-up Report [86] evaluated the disability status of infants born in 2008 only, and the combined 2004–2008 cohorts. Follow-up status was determined at age 18–24 months and information regarding death and NDI is provided for subgroups of children based on their gestational age. Therefore, we used these data to forecast expected trial results for our eligible subjects (24–27 weeks gestational age). In order to estimate the overall rate observed among treated neonates we have assumed that there will be no effect of treatment on death, but that Epo will lead to a decrease in the rate of NDI. If we assume a multiplicative reduction in the NDI rate of 0.45 then we expect a treated NDI rate of 12 % and an overall rate of death + NDI of 30.4 % as compared to the control rate of 40.4 %. Therefore, in order to obtain a target sample size we assumed: an overall control rate of 40 %, and an overall treated rate of 30 % corresponding to an overall treatment rate ratio of 0.75. Using the control and treated rates of 40 % and 30 % respectively leads to a sample size of 376 evaluated subjects per arm or a total evaluated sample size of 752 subjects in order to have 80 % power. We inflated the sample size by 20 % to account for correlation among multiple births (clustering) and potential loss to follow-up to arrive at a total enrollment target of 940 subjects.

#### Statistical analysis

A modified intent-to-treat (ITT) approach will be used [87], with all randomized infants who receive the first dose of study treatment included in the analysis. All pre-specified hypotheses will be tested using a two-sided type I error of 0.05 with no formal adjustment for multiple comparisons unless otherwise specified (such as with safety outcomes). Secondary analyses that focus on separate hypotheses will not require correction for multiple comparisons, but those analyses that use multivariate measures such as multiple brain image parameters would be corrected for multiple comparisons using standard methods.

Given that we anticipate enrollment of multiple births we require that all analyses properly account for the within-sibship correlation of outcomes. We will use Generalized Estimating Equations (GEE), which is a versatile regression approach for the analysis of discrete and continuous outcomes [88]. Use of “robust” standard errors will provide valid statistical inference and fully account for the clustering of data.

#### Data and safety monitoring

A data safety monitoring board (DSMB) created by National Institute of Neurological Disorders and Stroke (NINDS) will review the accruing data to ensure that the study is adequately enrolling and to ensure that there are no serious safety concerns. The research coordinators at each site monitor each subject daily for the presence of any complications. Serious adverse events are brought to the attention of an independent Medical Monitor, the DSMB, and IRB in writing. A potential risk that is unique to preterm infants is the risk of ROP [89]. In the published studies of preterm neonates receiving potentially neuroprotective doses of Epo, no difference has been noted between treatment and control groups [63, 90–92]. The DSMB will conduct formal interim safety monitoring analysis at approximately 25, 50, and 75 % and 100 % of target enrollment of *n* = 940 using O’Brien-Fleming [93] boundaries for death (net alpha = 0.05) and 10 serious adverse events (Bonferroni corrected alpha = 0.005).

## Discussion

Ample preclinical, and growing phase I and II data support the neuroprotective effects of Epo as an approach to improving the neurologic outcomes of ELGANS. In designing the PENUT Trial, we considered the issue of what constitutes a clinically significant effect size. Characteristics of trials that lead to changes in clinical practice include: the estimated magnitude of benefit; the number of subjects studied; and the risk of the therapy. Ibrahim et al. performed a 13-item web-based questionnaire asking 226 neonatologists what would convince them to adopt a new therapy in infants <28 weeks of gestation. The survey assumed no adverse results of treatment. The survey results suggest that a reduction of Bayley scores < 80 by 25 % of subjects would change behavior in 40 % of clinicians, while the same change in 50 % of subjects would persuade two thirds of neonatologists to adopt the intervention. A sample size of 200 per arm resulted in one third of neonatologists changing their behavior, while a sample size of 400 per arm resulted in two thirds changing behavior. The PENUT study is powered to detect a 45 % reduction in NDI, with a large enough sample size to be judged of sufficient quality to change practice for 64 % of clinicians surveyed [personal communication]. Epo neuroprotection is an exciting new approach to preterm neuroprotection, and if efficacious, will provide a much-needed therapy for this group of vulnerable infants.

## Conclusion

Current neuroprotective strategies include prenatal steroids, magnesium sulfate, delayed cord clamping, postnatal caffeine, breast milk, and avoiding postnatal growth retardation. Despite these measures, outcomes of extreme prematurity have not improved significantly over the last decades, with survivors remaining at significant risk of neurodevelopmental impairment. Erythropoietin has great potential to improve these outcomes. There is ample preclinical data showing beneficial effects of Epo on brain injury. Clinical trials to determine whether these preclinical findings translate to clinical improvement are ongoing.
